# Progestin-Primed Ovarian Stimulation is a non-inferior alternative to the GnRH Antagonist Protocol in patients undergoing assisted reproductive techniques: a retrospective study

**DOI:** 10.5935/1518-0557.20210057

**Published:** 2022

**Authors:** João Pedro Junqueira Caetano, Luciana Campomizzi Calazans, Leci Veiga Caetano Amorim, Leonardo Matheus Ribeiro Pereira, Erica Becker Sousa Xavier, Ana Luisa Menezes Campos, Bruna Barbosa Coimbra, Ricardo Mello Marinho

**Affiliations:** 1 Pro-Criar Reproductive Medicine Center (Eugin Group), Belo Horizonte, MG, Brazil; 2 Hospital das Clínicas - Universidade Federal de Minas Gerais, Medical Residency, Belo Horizonte, MG, Brazil; 3 Faculdade de Ciências Médicas de Minas Gerais, Belo Horizonte, MG, Brazil

**Keywords:** ovulation induction, progestin, pregnancy rate, reproductive techniques, assisted, *in vitro* fertilization

## Abstract

**Objective:**

To demonstrate the non-inferiority of Clinical Pregnancy Rates from Progestin-Primed Ovarian Stimulation compared to the GnRH Antagonist Protocol when the freeze-all and blastocyst transfer strategy is applied.

**Methods:**

A retrospective study included all IVF cycles performed at Pró-Criar Reproductive Medicine Center, Belo Horizonte, Minas Gerais, Brazil, between May 2018 and May 2019 using a GnRH antagonist analogue or oral progestins to block the LH peak in IVF/intra-cytoplasmic sperm injection (ICSI) cycles for infertility treatment.

**Results:**

The primary outcome of our study was Clinical Pregnancy Rate at the first ET (Blastocyst), which were 58.4% in the progestin group and 54.9% in the antagonist group (*p*=0.735), a finding consistent with most studies published to date using different progestins. The mean number of retrieved oocytes was 11 in the antagonist group and 9 oocytes in the progestin group (*p*=0.009). The fertilization rate was 80% for both groups (*p*=0.935). The rate of blastocyst formation per cycle was 50% in the antagonist group and 55.6% in the progestin group (*p*=0.106). The stimulation lasted a mean of 10 days in the two groups (*p*=0.403) and did not vary with patient age in either group. The gonadotropin dose used was higher in the antagonist group (2025 IU) than in the progestin group (1950 IU) (*p*=0.057). In addition, the blockade was effective: there was only one case of spontaneous ovulation, which corresponded to less than 1% of the cycles.

**Conclusions:**

Progestin-Primed Ovarian Stimulation is a non-inferior alternative to the GnRH Antagonist Protocol in patients undergoing assisted reproductive techniques. An incidence compatible with the 0.34 to 8% risk described in the literature for failure to control the premature LH surge in antagonist protocol cycles.

## INTRODUCTION

The use of assisted reproductive techniques (ART) has increased significantly in the last decade (Inhorn & Patrizio, 2015), becoming an important part of modern medicine, and playing a key role in family planning for many individuals (De Geyter, 2019).

The protocols used for ovulation induction during assisted reproductive treatments aim to obtain as many oocytes as possible in order to optimize the chances of treatment success. In general, gonadotropin-stimulating hormone (GnRH) antagonists or agonists are also included to avoid an early luteinizing hormone (LH) peak and, consequently, ovulation before oocyte retrieval. Traditionally, the use of such drugs was considered satisfactory for this function, although it carries high costs for the patient and causes discomforts associated with the injectable administration route (La Marca & Capuzzo, 2019).

In the current stage of ART development, in which embryo freezing is an increasingly common practice and provides good results, new options for controlling the LH peak and blocking ovulation can be considered (Kuang *et al*., 2015). The use of progestins has aroused interest in this regard, and the possible negative effect on the endometrium is no longer a concern, because now we can schedule embryo transfer (ET) for a later cycle (Massin, 2017; Yu *et al*., 2018).

Kuang *et al*. (2015) tested medroxyprogesterone acetate (MPA) for the prevention of premature luteinization in women undergoing in vitro fertilization (IVF) procedures, and they found results that were not inferior to those from the short agonist protocol (daily Triptorelin). Wang *et al*. (2016) conducted a randomized controlled trial (RCT) and showed that the use of MPA in the ovarian stimulation cycle can be effective and feasible without worsening pregnancy outcomes, and with a low incidence of ovarian hyperstimulation syndrome (OHSS) in women with polycystic ovary syndrome (PCOS). Iwami *et al*. (2018) and Yu *et al*. (2018) demonstrated that dydrogesterone could be used as a progestogen alternative for blocking the LH peak in IVF cycles, the latest being an RCT. There is more data demonstrating its long-term safety in pregnancy compared to MPA.

The study of pituitary suppression methods for inhibiting ovulation with the use of progestin, as addressed in the brief discussion above, has generated scientific interest and research in the field of assisted reproduction in recent years.

The aim of this study is to evaluate the non-inferiority of Clinical Pregnancy Rates in Progestin-Primed Ovarian Stimulation compared to the GnRH antagonist protocol.

## MATERIALS AND METHODS

This is a retrospective cohort analysis of 222 IVF/ICSI cycles performed at a single center, Pró-Criar Reproductive Medicine, Belo Horizonte, Brazil from May 2018 to May 2019.

The patients were divided into the Progestin group (n=112) and the Antagonist Group (n=110). During the study period, we ran 266 cycles, but 42 were excluded according to the following exclusion criteria: 1) women over 42 years old; 2) Cycles with fresh embryo transfer, 3) transfers of cleavage embryos (D2/D3); 4) embryos from cycles with preimplantation genetic screening; 5) cryopreservation of oocytes; 6) oocyte donation cycles; 7) cycles that produced embryos but without ET at the time of the analysis.

The primary outcome evaluated was the clinical pregnancy rate upon the first embryo transfer cycle. The secondary outcomes were the mean MII oocytes retrieved, fertilization rate, blastocyst formation rate, mean duration of stimulation and mean dose of gonadotropins.

The clinic's Ethics Committee approved the study, and all patients signed an informed consent form authorizing the use of data from their treatments in scientific studies.

### IVF/ICSI treatment protocol

Field experts chose the ovarian stimulation protocol. Controlled ovarian stimulation started after ultrasound on the 2^nd^-3^rd^ day of the spontaneous menstrual cycle or after the 4^th^-5^th^ day of pause from the combined oral contraceptive to evaluate the pituitary blockade (endometrium smaller than 5 mm and suppressed ovaries with absence of follicles larger than 10 mm).

The type of gonadotropin used did not follow a pattern, it was left to the discretion of the attending physician; both recombinant FSH (Gonal-F, Merck or Puregon, MSD) and hMG (Menopur, Ferring) were used at ranges from 150-300 IU daily. The initial and continuous gonadotropin dosages were adjusted according to patient age, baseline FSH level, body mass index (BMI), antral follicle count (AFC) and response to follicular growth in previous cycles. In the cycles that used the antagonist analogue to block the LH peak, the first ovulation-monitoring ultrasound was scheduled for the 5^th^ and 6^th^ stimulation days. Cetrorelix (Cetrotide, Merck) or Ganirelix (Orgalutran, MSD) daily was flexibly initiated when a follicle reached between 13 and 14 mm and was maintained until the day of ovulation trigger, which was performed with human chorionic gonadotropin (hCG) 10,000 IU (Choriomon-M, IBSA) or triptorelin acetate 0.2 mg (Gonapeptyl, Ferring), at the discretion of the attending physician. Oocyte pick-up (OPU) was performed 35 hours after the trigger injection.

In the cycles that used progesterone, 10 mg MPA 1x /day or 10 mg dydrogesterone (Duphaston, Abbott) 12/12 hour was started on the first day of ovarian induction and was maintained until the day of ovulation trigger, which could be performed with hCG (10,000 IU) or GnRH agonist analogue (0.2 mg of Triptorelin acetate), at the discretion of the attending physician. OPU was performed 35 hours after the trigger injection. Ovulation monitoring started between the 7^th^ and 8^th^ day for this group.

### Cryopreservation and thawing

We vitrified the embryos that reached the blastocyst stage between days 5-7 of development with good morphology. For the vitrification and thaw procedures, we used the medium from Ingamed, Brazil and the Kitazato Biopharma Co. protocol. For vitrification, we placed the embryos in an equilibrium solution VI-1 for 10 to 15 minutes, followed by 20 seconds in vitrification solution VI-2 until they were mounted on Cryo-Ingá (Ingamed, Brazil) straws with the minimum volume possible, followed by immediate immersion in liquid nitrogen. For thawing, the straw containing the embryos to be devitrified was immediately immersed in the warming solution DV-I for 1 minute and then transferred to diluting solution DV-II for 3 minutes, followed by a washing with buffer solution DV-III for 5 minutes, and a second washing with DV-III for 1 minute. Next, we placed the embryos in an incubator in a culture dish containing CSCM-C medium until the time of ET.

### Endometrial preparation and pregnancy confirmation

In all cycles, ET occurred after thawing, and the protocol was chosen at the discretion of the attending physician, as following: oral estradiol valerate (Primogyna, Bayer), 6 mg/d, started on the 1^st^ or 2^nd^ day of menstruation; Or prior blocking with GnRH analogue on the 21 day of the previous cycle, followed by oral estradiol valerate, 6 mg/d started after ultrasound confirmation of the blockade. Or natural cycle with hCG trigger or induced cycle with hCG trigger. Endometrial preparation was considered adequate when endometrial thickness was ≥7mm; estradiol was >200ng/ml and progesterone was <1ng/ml. All cycles used vaginal micronized progesterone (Utrogestan, Besins) at a dose of 400 mcg 12/12 hours (for 800 mcg/day). ET was performed 120 hours after the start of progesterone with pelvic ultrasound monitoring and the use of a soft catheter (Guardia Access Catheter, Cook Medical). The β-hCG level was measured 9 days after ET. Biochemical pregnancy was confirmed when the β-hCG level was >30 IU/L. Clinical pregnancy was confirmed based on the detection of gestational sacs by means of endovaginal ultrasound, 3 weeks after ET

### Statistical analysis

The qualitative variables were expressed as absolute and relative frequencies, and the quantitative variables were expressed as the mean±standard deviation (sd), when normally distributed and by median±interquartile range (IQR) when otherwise. The quantitative variables were subjected to the Shapiro-Wilk normality test. The association between qualitative variables was evaluated using the chi-square test of independence or the Fisher's exact test, when the Chi-square test could not be applied. To compare quantitative variables between the antagonist and progestin groups, we used the Student's t-test for variables with normal distribution, and the Wilcoxon Mann-Whitney test when otherwise, both for independent samples. We ran the analyses using the free program R version 3.5.1, considered significant at *p*<0.05.

### Sample size

The sample size required to test the difference in clinical pregnancy rate in the progestin and antagonist protocols was calculated using the following formula:


$$\mathrm{nProgest}={\left(\frac{z_{1-a/2}+z_{1-\beta }}{\mathrm{pProgest}-\mathrm{pAntag}}\right)}^{2}\left(\frac{\mathrm{pAntag}\left(1-\mathrm{pAntag}\right)}{\pi }+\mathrm{pProgest}\;\left(1-\mathrm{pProgest}\right)\right)$$

e


$$n_{\mathrm{Antag}}=\tau n_{\mathrm{Progest}}$$

in which Z_1-α/2_ and Z_1-*β*_ denote percentiles of the standard normal distribution associated with the test's significance and power, respectively; *pProgest* and *pAntag* are the proportions of clinical pregnancy rates from a previous study. Considering a 5% significance level, a minimum power of 80% and *τ* = 1, at least 100 women in the progestin group and 100 women in the antagonist group were required to test the difference in clinical pregnancy rates.

## RESULTS

We performed 266 IVF/ICSI cycles between May 2018 and May 2019; of those, 222 cycles (83.5%) were included in the study, 110 in the GnRH Antagonist Protocol group and 112 in the Progestin Protocol group. Table 1 summarizes the demographic characteristics of the patients in the two groups analyzed. Age, BMI and duration of infertility were equally distributed between the two groups. Basal day 3 FSH was higher in the Progestin group and AFC higher in the Antagonist group.

**Table 1.** Characteristics of the women included in the sample according to the ovarian stimulation protocol used.

Characteristic	Antagonist (n=110)	Progestin (n=112)	*p*-value
Age (years) *	35±4	35±7.25	0.658^W^
<34	52 (47.3%)	54 (48.2%)	
35 to 37	32 (29.1%)	29 (25.9%)	
38 to 40	21 (19.1%)	18 (16.1%)	
41 and 42	5 (4.5%)	11 (9.8%)	
BMI* (kg/m^2^) *	23.18±4.86	23.67±5.01	0.497^W^
Duration of infertility (months)**	36±29	36±36	0.285^W^
AFC (n) *	19±13.25	16±9.75	<0.001^W^
Serum FSH* IU/L **	6.96±1.98	7.87±2.56	0.011^T^

* Data presented as median ± IQR

** Data presented as median ± SD

W Wilcoxon Mann-Whitney,

T t-test (both for independent samples)

### Ovarian stimulation outcomes

Table 2 shows that the median duration of stimulation, gonadotropin dose used and fertilization rate did not differ significantly between the two groups. The stimulation lasted a mean of 10.00±1.00 days in the two analyzed groups (*p*=0.403). The gonadotropin dose used was higher in the antagonist group (2025±586.25) than in the progestin group (1950±581.25) (*p*=0.057).The fertilization rate was 80.00±20.68 for the antagonist group and 80.00±30.83 for the progestin group (*p*=0.935).

**Table 2.** Clinical outcomes according to ovarian stimulation protocol.

Characteristic	Antagonist	Progestin	*p*-value
Stimulation duration (days)*	10.00±1.00	10.00±1.00	0.403^W^
Gonadotropin dose (UI) *	2025±586.25	1950±581.25	0.057^W^
Number of MII oocytes *	11.00±8.00	9.00±6.00	0.009^W^
Fertilization rate (%)*	80.00±20.68	80.00±30.83	0.935^W^

* Data presented as median ± IQR

W Wilcoxon Mann-Whitney test

There was a significant difference in the median number of retrieved oocytes. The median number of retrieved oocytes was 11.00±8.00 in the antagonist group and 9.00±6.00 in the progestin group (*p*=0.009), as per shown in Table 2.

**Figure 1:**
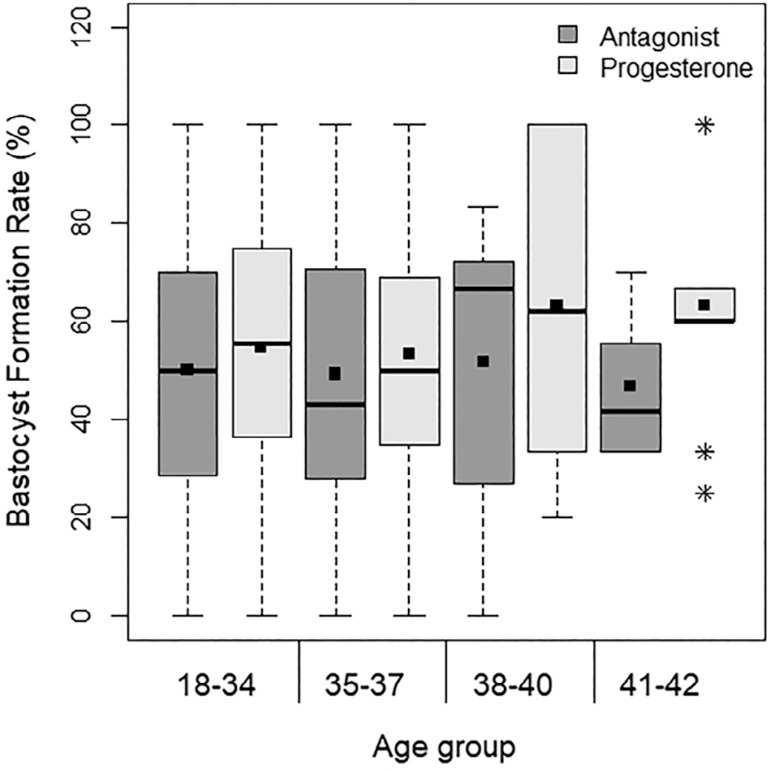
Distribution of the blastocyst formation rate (%), according to age group and ovarian stimulation protocol The means are presented as squares.

The rate of blastocyst formation per cycle was 50.00%±41.79 in the antagonist group and 55.60%±38.64 in the progestin group (*p*=0.106) (Table 3). The rate of blastocyst formation according to age groups is shown in Figure 1. It did not differ significantly between any of the groups (Table 3).

**Table 3.** Blastocyst formation rate.

Characteristic	Antagonist	Progestin	*p*-value
Blastocyst Formation Rate per cycle (%)*	50.00±41.79	55.60±38.64	0.106^W^
18 - 34 y.o. **	50.12±24.29	54.58±26.21	0.372^T^
35 - 37 y.o . **	49.18±25.14	53.37±26.31	0.535^T^
38 - 40 y.o *	66.70±45.60	61.90±61.74	0.266^W^
41 - 42 y.o . **	46.78±15.84	63.18±25.26	0.163^T^

* Data are presented as median ± IQR

** Data are presented as median ± SD

W Wilcoxon Mann-Whitney test

T t-test (both for independent samples)

### Clinical Pregnancy rate - primary outcome

**Figure 2:**
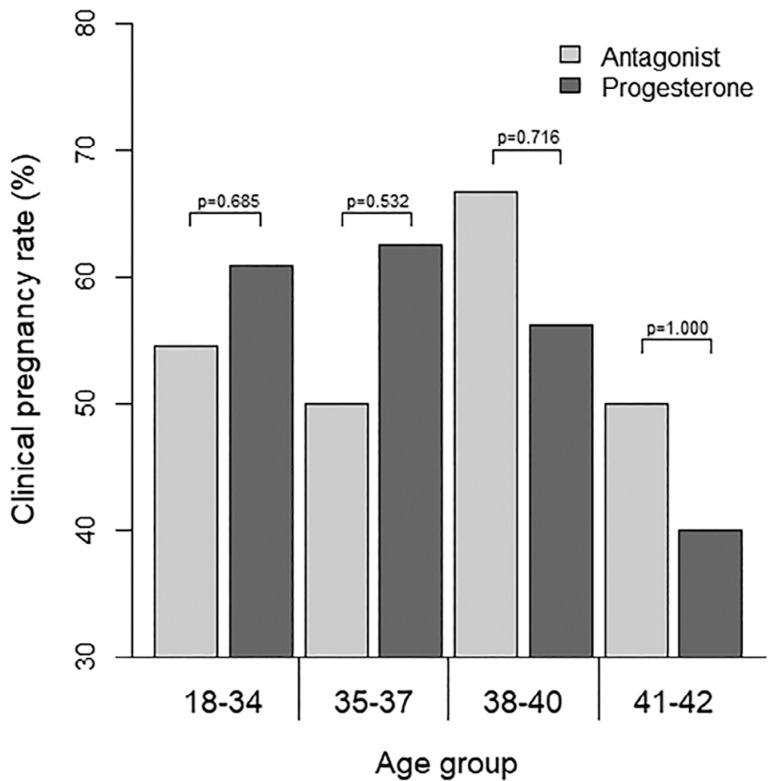
Distribution of the clinical pregnancy rate (%) according to age group and ovarian stimulation protocol *p*-values refers to Chi-square or Fisher's exact test.

Table 4 shows the IVF outcomes in Clinical Pregnancy Rate (CPR) in both groups. The CPR did not differ significantly between the two groups; it was 58.5% in the progestin group and 54.9% in the antagonist group (*p*=0.735). CPR was also calculated for different age subgroups, as shown in Figure 2: 18-34 years old; 35-37 years old, 38-40 years old and 40-42 years old. CPR did not differ significantly between the groups (Table 4).

**Table 4.** Clinical Pregnancy Rate (%) at the first ET according to age group.

Characteristic	Antagonist (n=110)	Progestin (n=112)	*p*-value
CPR	50 (54.9%)	59 (58.4%)	0.735^C^
18 to 34 years	24 (54.5%)	31 (60.8%)	0.685^C^
35 to 37 years	14 (50.0%)	15 (62.5%)	0.532^C^
38 to 40 years	10 (66.7%)	9 (56.2%)	0.716^F^
41 and 42 years	2 (50.0%)	4 (40.0%)	1.000^F^

C Chi-square test of independence

F Fisher’s exact test

## DISCUSSION

Our results were not inferior in the incidence of premature LH surge with oral progestins compared to blockade with antagonist in cycles with blastocyst freeze-all.

Whenever a new protocol is incorporated into the medical routine, there is concern that it may have some impact on patients' reproductive outcomes. The duration of stimulation and the gonadotropin doses used in the studied population did not differ between the groups, and there was a statistically superior production of mature oocytes (MII) in the group that used the Antagonist.

Laboratory outcomes, such as fertilization rate and blastocyst formation rate, were similar between the groups. The primary study outcome, clinical pregnancy rate at the first thawed blastocyst transfer, was comparable between the groups, a finding consistent with most studies published to date using different progestins (Albertini, 2015; Barnhart, 2014; Borm & Mannaerts, 2000; Chen et al., 2017; Wang et al., 2016; Wong et al., 2014; Yu et al., 2019). The only study to date that showed unfavorable pregnancy rates with the use of progestins was conducted in oocyte donors, and the embryos were transferred to non-randomized recipients (Cobo et al., 2012). In addition, the blockade was effective: there was only one case of spontaneous ovulation, which corresponded to less than 1% of the cycles, an incidence compatible with the 0.34 to 8% risk described in the literature for failure to control the LH peak in antagonist protocol cycles (De Geyter, 2019; Inhorn & Patrizio, 2015; Iwami et al., 2018; Kuang et al., 2015). We subdivided the populations by age group to assess whether any group would be affected by the use of this protocol, and there were no such effects.

The use of progestin to block the LH peak has the advantages of ease of oral administration and lower cost compared to antagonist analogues; additionally, it allows more flexible ovulation monitoring and is therefore more comfortable for the patient. There could be a concern regarding the need to transfer frozen embryos in a subsequent cycle due to the detrimental impact of early endometrial exposure to progesterone. However, we did not consider this a limiting factor, because the evolution of cryopreservation techniques has led to comparable results for frozen embryo cycles and fresh ET (Massin, 2017; Reichman *et al*., 2014; Roque, 2015; Shapiro *et al*., 2009; Van Wely *et al*., 2003). The freeze-all strategy also enables the embryos to be transferred into a more physiological uterine environment (Wang *et al*., 2016). Although this was not a randomized controlled trial, and the choice of protocol depended on the attending physician, statistical analysis showed that the studied populations were similar, with a similar mean age, BMI, duration of infertility and distribution of causes of infertility between the groups.

Although the gonadotropin used was not standardized, systematic reviews have found no evidence that the choice of gonadotropin influences the outcome of assisted reproductive treatments (Wong *et al*., 2014). The gonadotropin doses used followed the clinical guidelines, and there was no difference between the groups. There was a significant difference only for AFC and baseline FSH, suggesting a better ovarian reserve in patients in the antagonist group, which could be an advantage over the progestin group. In addition, the antagonist group produced a greater number of MII oocytes per cycle. Nevertheless, this difference was not present in the blastocyst formation rate and CPR, showing that it had no clinical impact.

During the study period, the commercialization of MPA in Brazil was suspended, and it was replaced in our clinic by dydrogesterone (Duphaston, Abbott), so that two different progestins were used in the cycles studied. A previous study (Beguería *et al*., 2019) showed similar efficacy for the two progestins. Other studies have also shown similar results with the use of Utrogestan (Wang *et al*., 2018). We understand that the blocking mechanism is the same and that there would be no impact on oocyte quality and therefore no difference according to the type of progesterone used.

## CONCLUSION

The study demonstrated the non-inferiority in Clinical Pregnancy Rates in Progestin- Primed Ovarian Stimulation compared to the GnRH Antagonist Protocol when the freeze-all and blastocyst transfer strategy is applied.

As well as similar secondary outcomes, such as blastocyst formation rate, duration of stimulation, gonadotropin dose and fertilization rate, indicating that the use of progestin did not affect the quality of the oocytes obtained. Progestins are an excellent alternative to antagonists because it is an easily accessible medication; it is administered orally and has lower cost. In addition, it allows greater flexibility to initiate ultrasound monitoring, thus facilitating treatment management for patients and physicians. On the other hand, it may be used only on freeze-all programs, not allowing fresh transfers. The protocols using progestins to block LH surge have the potential to be used not only for special situations as oocyte donation programs, social and oncology preservation and dual stimulation (duostim), but even in a regular basis into the clinics. Randomized controlled trials should be conducted to confirm the viability of this regimen, the ideal dose, the types of progestins to use, and long-term safety.
